# Practical Points in the Management of Some of the Diseases of Children

**Published:** 1891-09

**Authors:** 


Practical Points in the Management of Some of the Diseases of Children. By I. N. Love, M. D., Professor of Diseases of Children, Clinical Medicine and Hygiene in the Marion-Sims College of Medicine, St. Louis ; Consulting Physician to the City Hospital, St. Louis, etc., etc. The Physicians' Leisure Library. Detroit : George S. Davis. 1891. Price, cloth, 50 cents ; paper, 25 cents.

There is no more important department of medicine than that which is concerned in the management of the diseases of children. By judicious care in the early weeks or months of child life, many of the maladies and deformities that mar symmetry or affect health in after years may be avoided. It is a field in which an especial aptitude is required to successfully conduct practice, and only a few men seem to be gifted with the tact requisite to do so. The author of this little volume is eminently one of those men who may be termed a master in the department of pediatrics, and who never writes with an uncertain or diffident pen. If we had space at hand it would be interesting to quote at length from Dr. Love's admirable book. We cannot refrain, however, from bringing out one point, which is of such particular importance as to demand attention from every person concerned in the care of infants. Dr. Love says on page thirty-nine :

Let us not forget that in guarding the skin of children as well as adults, we should keep in proper condition the alimentary canal primarily, and urge upon those under our care the importance of the hygiene of the skin, bathing at least once a day with tepid water and a proper kind of soap. Dr. Merrill B. Ricketts, of Cincinnati, at a recent meeting of the Mississippi Valley Medical Association, presented a paper upon skin diseases resulting from the abuse of soaps, wherein he made very strong points. Undoubtedly there is not proper discretion exercised on the part of the household in the selection of soaps for domestic use. White castile soap is a good all-around soap, but one of the best for use in the nursery is Pears' soap, and in spite of the fact that somebody may benefit by this special commendation, I am ready to maintain the position, and present in justification the fact that probably the highest authority in the world upon the skin, Sir Erasmus Wilson, has publicly taken the same position.

We regard this one of the most important publications in the Leisure Library series, and should recommend every physician to possess it, for it is equally as useful in the household as in the medical library.

